# Saffron: An Old Medicinal Plant and a Potential Novel Functional Food

**DOI:** 10.3390/molecules23010030

**Published:** 2017-12-23

**Authors:** María José Bagur, Gonzalo Luis Alonso Salinas, Antonia M. Jiménez-Monreal, Soukaina Chaouqi, Silvia Llorens, Magdalena Martínez-Tomé, Gonzalo L. Alonso

**Affiliations:** 1 Cátedra de Química Agrícola, E.T.S.I. Agrónomos y de Montes, Universidad de Castilla-La Mancha, Campus Universitario, 02071 Albacete, Spain; MariaJose.Bagur@uclm.es (M.J.B.); chaouqi89@hotmail.fr (S.C.); 2 Department of Food Science, Universidad de Murcia, Regional Campus of International Excellence, Campus International de Excelencia Regional “Campus Mare Nostrum”, CIBERobn, ISCIII, 30100 Murcia, Spain; antoniamjimenez@um.es (A.M.J.-M.); mmtome@um.es (M.M.-T.); 3 Heart Failure Unit, Department of Cardiology, Hospital Ramon y Cajal, 28034 Madrid, Spain; gonzalol.alonso@gmail.com; 4 Laboratory of Materials, Environment and Electrochemistry, Faculty of Science, Ibn Tofaïl University, P.O. Box 242, 14000 Kénitra, Morocco; 5 Department of Medical Sciences, School of Medicine and Regional Centre for Biomedical Research (CRIB), University of Castilla-La Mancha, 02008 Albacete, Spain; Silvia.Llorens@uclm.es

**Keywords:** saffron, crocin, crocetin esters, safranal, picrocrocin, nutraceutical, therapeutic properties, functional food

## Abstract

The spice saffron is made from the dried stigmas of the plant *Crocus sativus* L. The main use of saffron is in cooking, due to its ability to impart colour, flavour and aroma to foods and beverages. However, from time immemorial it has also been considered a medicinal plant because it possesses therapeutic properties, as illustrated in paintings found on the island of Santorini, dated 1627 BC. It is included in Catalogues of Medicinal Plants and in the European Pharmacopoeias, being part of a great number of compounded formulas from the 16th to the 20th centuries. The medicinal and pharmaceutical uses of this plant largely disappeared with the advent of synthetic chemistry-produced drugs. However, in recent years there has been growing interest in demonstrating saffron’s already known bioactivity, which is attributed to the main components—crocetin and its glycosidic esters, called crocins, and safranal—and to the synergy between the compounds present in the spice. The objective of this work was to provide an updated and critical review of the research on the therapeutic properties of saffron, including activity on the nervous and cardiovascular systems, in the liver, its antidepressant, anxiolytic and antineoplastic properties, as well as its potential use as a functional food or nutraceutical.

## 1. Chemical Composition of Saffron

The dried stigmas of the *Crocus sativus* L. flower constitute what is known as saffron spice. The Spanish Food Code [1] indicates that it will not exceed the following maximum values: 15% moisture and volatile matter; 8% ashes; 2% silica; 6% raw fibre; between 3.5 and 14.5% of ethereal extract. Saffron is recognised for providing colour, flavour and aroma to foods and drinks when added. Due to the unique organoleptic attributes of saffron, and the difficulties involved in its cultivation, harvesting and handling, it has high value and is considered the most expensive spice in the world. For this reason, and because of stigmas’ colour, it is known as “red gold” [2]. Saffron is regarded as high quality when the concentration of its compounds which lend organoleptic characteristics are elevated. In addition, the quality of the product is also defined by the absence of other substances, such as any other type of food colouring, and by a low quantity of other certain elements, such as flower remains, soil and insects [3]. The chemical composition of saffron has been studied in detail by various authors [4]. Chemical analysis has shown the presence of more than 150 components in the stigmas of saffron [5]. In addition to the three main components in saffron (crocetin esters, picrocrocin and safranal), it also contains other carotenoids, carbohydrates, raw fiber, proteins, fats, anthocyanins, flavonoids, vitamins (riboflavin and thiamine), minerals and many other elements which confer nutritional properties and are beneficial to health [6,7]. No nutritional parameter is used in determining the commercial quality of saffron, except moisture to prevent weight fraud.

The most important use for saffron is in food, where it is valued for its colouring, flavouring and aromatizing in the production of some traditional dishes. In addition to its use as a spice, saffron has long been considered a medicinal plant for its therapeutic properties [8]. In frescoes found on the island of Santorini, dating back to 1627 BC, it is possible to observe an offering of some type of *Crocus* stigmas (*C. sativus* or *C. cartwrightianus*) being made to the goddess Thera. The investigations of Ferrence and Bendersky [9] attributed this to the healing properties of saffron. Indeed, saffron was included in the Catalogues of Medicinal Plants and in the European Pharmacopoeia from the 16th until the 20th centuries, as part of numerous pharmaceutical preparations [10]. However, the medicinal and pharmaceutical use of saffron disappeared with the advent of synthetic chemistry-derived drugs. More recently, however, scientific interest in revisiting saffron’s known bioactivity has been growing [11,12]. The bioactive properties observed in recent scientific publications are attributed to the main components, mentioned above, but also to the synergistic activity of all the compounds present in the spice.

### 1.1. Compounds Responsible for the Colour

Carotenoids are responsible for the intense colour that saffron provides to aqueous solutions. The main carotenoid of saffron was first isolated by Aschoff in 1818, it was called crocin, derived from the word “crocos”, which means saffron in German. Decker [13] showed its glycosidic nature and Karrer and Solomon [14,15,16] established its structure and molecular formula. The name according to the International Union of Pure and Applied Chemistry (IUPAC) is bis[(2*S*,3*R*,4*S*,5*S*,6*R*)-3,4,5-trihydroxy-6-[[(2*R*,3R,4*S*,5*S*,6*R*)-3,4,5-trihydroxy-6-(hydroxymethyl)oxan-2-yl]oxymethyl]oxan-2-yl] (2*E*,4*E*,6*E*,8*E*,10*E*,12*E*,14*E*)-2,6,11,15-tetramethylhexadeca-2,4,6,8,10,12,14-heptaenedioate, but it has also been named as digentiobiosyl 8,8′-diapocarotene-8,8′-dioate [17].

**Figure 1:**
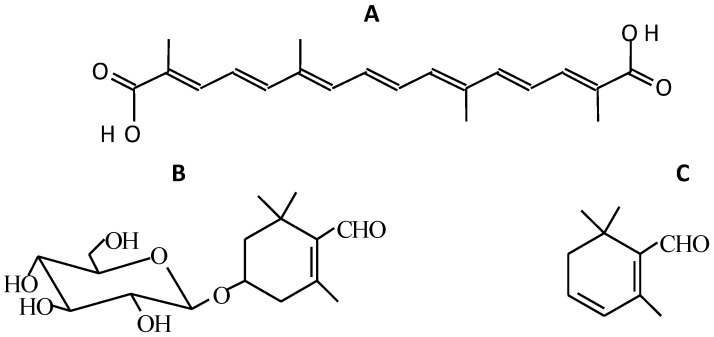
Chemical structures of crocetin (**A**), picrocrocin (**B**) and safranal (**C**).

**Figure 2:**
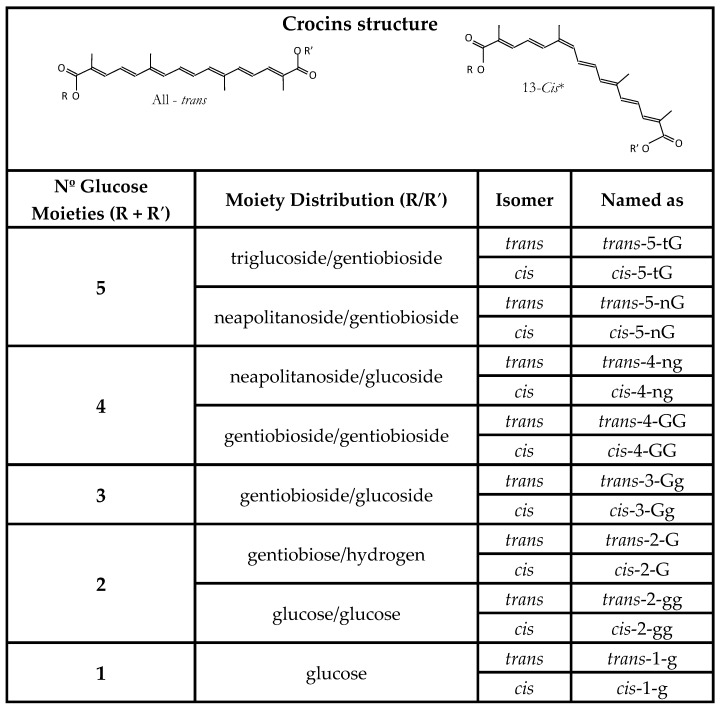
Simplified name of the glycosidic esters, crocins, of the carotenoid crocetin introduced by Carmona and coworkers [22]. Meaning of the abbreviations: t is triglucose; G is gentiobiose; n is neapolitanose; g is glucose.

Crocetin (Figure 1A) is the dicarboxylic carotenoid that, after glycosylation, receives the name of crocin. The crocins present in the aqueous extracts of saffron pool together and, at high concentrations, they undergo aggregation [18]. In 1982, Pfander isolated six crocins [19], while, after two years, Speranza identified the *cis-* and *trans*-isomers of crocins by high performance liquid chromatography (HPLC) and UV-visible spectrophotometry (UV-V) [20]. In 1995, Tarantilis identified numerous crocins [21] and, in 2006, Carmona and co-workers recognized four more crocins, and they simplified the glycosidic esters name to that which is shown in Figure 2 [22].

Interest in objectively establishing the quality of saffron has led to the development of numerous extraction and analysis methods to separate, identify and quantify the esters of crocetin. The method developed by Corradi and Micheli [23] is based on measuring the absorption at 440 nm of an aqueous extract of saffron by UV-vis spectrophotometry. This is because the esters of glycosidic crocetin reach their maximum absorption at this wavelength, making it possible to determine the total number of carotenoids and their colouring power. However, to separate, identify and quantify each of the different glycosidic esters of crocetin present in saffron, methods were developed based on high performance liquid chromatography with a UV-vis detector (HPLC-UV-vis) [19,20], thin layer chromatography (TLC) [24], and HPLC with aligned diode detectors and mass spectrometry (HPLC-DAD-MS) [21,22]. Until recently, quantifying crocetin has been quite complex due to the non-availability of commercial patterns [25] and the phenomenon of molecular self-aggregation, which occurs in concentrated aqueous dilutions [18]. Nowadays, they can be determined since high purity commercial standards exist in *trans*,*trans*-4-GG and *trans*-3-Gg crocetin ester forms [26]. The content of glycosidic esters of crocetin in the spice varies depending on the quality, but may reach very high percentages of between 25 and 35% on a weight basis in dried saffron [26]. This high concentration of carotenoids may be responsible for saffron’s bioactive properties.

### 1.2. Compounds Responsible for the Bitter Taste

Picrocrocin (C_16_H_26_O_7_, 4-(β-d-glucopyranosyloxy)-2,6,6-trimethyl-1-cyclohexene-1-carbox-aldehyde, Figure 1B) is the substance responsible for the bitter taste of saffron. Its presence in saffron can constitute up to 26% of the dry matter [27]. The structure of picrocrocin was established by Khun and Winterstein in 1934 [28]. Picrocrocin is the precursor of safranal and has only been identified in the *Crocus* genus, whose only edible species is the *C. sativus*. The kinetics of picrocrocin degradation in aqueous extracts of saffron upon thermal treatment from 5 °C to 70 °C was determined by Sánchez and coworkers [29]; picrocrocin was found to be more stable than the crocetin esters.

### 1.3. Compounds Responsible for the Aroma

More than 40 compounds related to the aroma of saffron have been identified, the major compound being safranal (2,6,6-trimethyl-1,3-cyclohexadiene-1-carboxaldehyde, Figure 1C) [30]. In the world’s highest quality saffron, “Azafrán de La Mancha”, safranal represents more than 65% of the total aroma components [31].

During the process of dehydration, manipulation and storage of saffron, safranal is generated by the hydrolysis and dehydration of picrocrocin. It is determined in accordance with International Organisation for Standardization (ISO) 3236 standard [2] by measuring the absorbance at 330 nm from an aqueous extract of saffron. However, this technique has limitations, as the mentioned wavelength also absorbs *cis* isomers derived from the crocetin [32]. Other methods used are HPLC [26] for the independent quantification of the crocetin esters, picrocrocin and safranal using only a saffron solution prepared according to the ISO 3632 and gas chromatography (GC) for the determination of all volatile compounds [33].

### 1.4. The Need to Analyze the Main Components of Saffron

Saffron is frequently confused with parts of other plants, which are also regarded as spices, although they are not capable of providing saffron’s characteristic color, flavor and aroma to food. This occurs with safflower and marigold petals when the saffron is presented as whole stigmas and with turmeric when it is sold ground [4]. To detect the falsification of saffron when fragments from other species are added to the stigmas of the crocus flower, it is necessary to know the shape of the stigmas of the flowers of *C. sativus*. The best way to detect falsification or adulteration is to analyze the content of the main metabolites (crocins, picrocrocin and safranal) with the methods described above, as well as requiring that there is minimum content of these compounds in the spice.

## 2. Bioaccessibility, Bioavailability and Bioactivity of Saffron’s Compounds

The bioaccessibility of a chemical compound in a foodstuff is defined as the quantity or fraction that is released from the matrix of the foodstuff in the gastrointestinal tract to become available for absorption [34,35]. The concept of bioavailability refers to the proportion of a nutrient contained in a foodstuff that is absorbed for use and storage by the body [36]. The term bioavailability includes bioactivity when the nutrient reaches the target tissue and the physiological response occurs.

The pharmacokinetics of the carotenoids most widespread in nature are known. Due to their lipophilic character, after their release from the foodstuff matrix, they are absorbed through the intestinal cells by passive diffusion, and are incorporated into chylomicrons without being modified before secretion into the bloodstream [37,38].

However, the major carotenoids in saffron have the peculiarity of being soluble in water because they are glycosidic esters. Although there are many studies that show their various biological activities in humans, the form of administration, absorption and metabolism of these carotenoids is yet to be discovered [39]. From the pharmacokinetic point of view, we assume that the crocins are not absorbed after oral administration, but are largely hydrolyzed to crocetin in the intestinal tract [40]. The first data on the bioaccessibility of esters of crocetin and picrocrocin from an aqueous extract of saffron was presented by Kyriakoudi and coworkers [41], who indicated that approximately 50% and 70%, respectively, were bioaccessible under in vitro gastrointestinal digestion conditions. A subsequent study suggested that, despite this high degree of bioaccessibility, the quantities transported (0.5% and 0.2%, respectively) are ten times lower than crocetin [42]. The bioavailability of the glycosylated crocin extract of saffron (free of crocetin) was detected by Lautenschlager and coworkers [43]. In vitro studies have shown that the crocins in saffron are probably not bioavailable in the systemic compartment after oral consumption. This is because they are hydrolyzed rapidly, mainly by enzymes in the intestinal epithelium and, to lesser extent, by the intestinal microbiota, to deglycosylated *trans*-crocetin, which is absorbed by passive diffusion through the intestinal mucosa layer [43]. The oral administration of crocin leads to a 56–81 times higher concentration of crocetin in rat serum than the oral administration of crocetin, which is interesting given that the pharmacological effect is attributed to the *trans*-crocetin isomer [44].

*trans*-Crocetin is the only active metabolite that has been shown to be capable of crossing the blood–brain barrier and reach the central nervous system (CNS), regardless of whether pure crocetin or saffron extract is administered. Yoshino and coworkers [45] showed that this occurs 90 min after the pure crocetin has been orally administered to rats. Similarly, Linardaki and coworkers [46] detected crocetin in the mice’s brains after the intraperitoneal administration of saffron extract.

The pharmacokinetics of *trans*-crocetin has been validated in animal models [47,48,49] and in the plasma of healthy volunteers [50]. Mohamadpour and coworkers [51] proposed a quick and sensitive method to determine crocetin in human serum, which will be useful for future clinical pharmacokinetics studies. Unlike other carotenoids, crocetin is absorbed considerably more rapidly, being detected in plasma one hour after administration and peaking around 4 h later. Pharmacokinetics studies performed in other C40 carotenoids, such as carotene, lutein and lycopene, have shown that after oral administration they require greater time to reach peak concentrations [50]. These differences could be explained because *trans*-crocetin is more hydrophilic and smaller than other carotenoids (C20). For their part, Asai and coworkers [47], based on a study on mice, proposed a metabolic pathway model for *trans*-crocetin and crocin. This research group showed that after oral crocins administration, the crocin is hydrolyzed to *trans-* and *cis*-crocetin. Subsequently, the *trans*-crocetin may be partially conjugated with mono and diglucuronides in the intestinal mucosa and/or in the liver and thus, via the portal vein, it can reach the bloodstream.

There is evidence that reactive oxygen substances (ROS) generated by muscular exercise are one of the causes of physical fatigue [52]. In a study on the anti-fatigue effect of crocetin, Mizuma and coworkers [53] ascertained that its intake improved performance when taken 4 h before a physical fatigue-inducing task. Kanakis and coworkers [54] studied the interaction of crocetin with serum albumin in humans and they found that the protein–ligand interaction forms a weak bond, which explains why crocetin tends to be easily distributed to the tissues of the body. Umigai and coworkers [50] also observed that the pharmacokinetics of crocetin was proportional to the dose. The above studies show that, unlike other carotenoids, crocetin is rapidly absorbed and can be detected in the plasma within an hour of administration. The mean maximum concentration is around 4 h, which decreases gradually, with crocetin being detectable up until a limit of 24 h.

## 3. Relationship between Bioactivity and Antioxidant Capacity of Saffron

Oxidative stress, resulting from an imbalance between radical-generating and radical-scavenging systems, is implicated in more than 100 different diseases, including several types of cancer, heart and vascular diseases, obesity and neurodegenerative disorders [55]. Amongst these, neurodegenerative disorders are Alzheimer’s, Parkinson’s, Huntington’s and Multiple Sclerosis (MS) [56,57].

In the last decade, numerous scientific reviews have highlighted the biomedical and pharmacological properties of saffron or its metabolites, including a lot of bioactions related to the disorders of the nervous, blood, cardiovascular, respiratory, renal, digestive and endocrine systems. These bioactions include the enhancement of oxygen diffusivity, increment of ocular blood flow, inhibition of tumor cell proliferation, chemoprevention, and protective effects against atherosclerosis, hepatotoxicity and hippocampal disorders, amongst others [8,11,58,59]. Saffron and its constituents—crocin and safranal—have been shown to be powerful ROS, which is the cause of some of the spice’s healthy effects [54,60,61,62,63,64].

For several decades, the possible beneficial role of saffron or glycosidic esters of crocetin in the prevention and development of various chronic diseases has been considered. The results obtained, which are broadly in line with previous knowledge, have allowed advances in research, with carotenoid components being identified as responsible for different beneficial effects when introduced into the diet [65]. The spice’s high antioxidant capacity explains most of its preventive or healing properties in relation to chronic and degenerative diseases [7,66,67].

The beneficial effects of crocetin esters have been demonstrated on many body systems: gastrointestinal, cardiovascular, endocrine, reproductive and immune, among others, the CNS being the most studied [7]. Although many of these studies relate the effects to the antioxidant-rich nature of the carotenoids in saffron, the action mechanisms have not been sufficiently studied. Nam and coworkers [68] suggested that crocin and crocetin may have a neuroprotective effect because of their anti-inflammatory action in microglial cells, as tested in rat brains, accompanied by a reduction in neurotoxic molecules such as TNF-α, interleukin-1β and intracellular ROS. The restoration of a redox balance in brain tissues can be a good therapeutic strategy to limit neuro-inflammation and consequently tissue oxidative damage [69].

As stated above, safranal is generated from picrocrocin during the dehydration, manipulation and storage of saffron. Picrocrocin has been shown to have antiproliferative activity in human cell cancers [42]. However, the bioactivity attributed to the deglycosylated derivate of picrocrocin and safranal presents great variability and is less well known than that of crocin [42]. There are studies that attribute specific effects to safranal, especially as an antidepressant, inducer of satiety [70] and as an anticonvulsant. Crocins do not have such effects [71,72]. Safranal combats oxidative stress in neurons, scavenging free radicals [54].

Some authors attribute antioxidant properties indiscriminately to any of the components of saffron [60,61,62]. Others have noted that safranal has no action on certain effects such as sexual activity, attributing them to the aqueous extract of saffron and crocin [73].

In most studies, there is no thorough characterization of the composition of the saffron supplied, so it is not possible to confirm that the crocetin esters are responsible for such action, and rather the effects may be produced by safranal, picrocrocin or other compounds.

## 4. Therapeutic Properties of Saffron

Saffron extracts and tinctures have been used for centuries in traditional medicine for the treatment of different syndromes and diseases [4]. Some of these uses have been antispasmodic, eupeptic, sedative, carminative, diaphoretic, expectorant, stomachic, stimulant, aphrodisiac, emmenagogue and abortifacient. It was previously widely used in the treatment of genital diseases, and menstrual regulation and relief. The spice’s abortive action was well known in the Middle Ages, during which it was also used by midwives in deliveries for the sedative and antispasmodic action of saffron [4]. It has also been used to treat eye diseases, heal wounds, fractures and joint pain and for many other uses, leading to Pliny the Elder describing it as a kind of panacea in his Naturae Historiarum XXXVII [4].

The strong interest in the potential biomedical applications of this spice are precipitated by the great public health problems that currently result from the disorders saffron treats.

### 4.1. Activity of Saffron in the Central Nervous System (CNS) and the Peripheral Nervous System

Pharmacological effects on the CNS have been attributed to saffron and its metabolites, including a biological action on memory and learning, neurodegenerative diseases, depression and anxiety, among others. The biological activity of these molecules in this system have been studied for over two decades [74,75,76,77,78,79,80]. These works are some examples of the profusion of literature supporting this bioactivity. Among the numerous effects shown on the CNS, we draw attention to the following:

#### 4.1.1. Effect on Memory and Learning

Neurodegenerative disorders are often associated with alterations of memory and learning [7]. Many people with Multiple Sclerosis (MS) experience a decrease in memory, although the risk factors for this have not been identified [81]. Abe and coworkers [82] attributed to crocetin esters an antagonistic action towards the potentiation induced by ethanol in the neurons of the hippocampus. They also suggested a possible mechanism of action on *N*-methyl-d-aspartate (NMDA) receptors, which are receptors of the neurotransmitter glutamate, with actions in neuronal plasticity and memory. Likewise, by studying the protective effects of an extract of saffron and its glycosidic crocetin esters, the authors concluded that it could prevent the impairment of learning and memory, as well as prevent damage by oxidative stress in the hippocampus as a result of chronic stress.

Other publications by Abe and Saito [76] and Sugiura and coworkers [75] also highlight the action of saffron extract or its active constituents in the improvement of memory and learning skills.

In vivo studies show that they also have an affirmative effect on learning deficits and are involved in the underlying mechanisms of recognition and spatial memory [83]. Treatment with saffron extract for seven consecutive days in a study conducted in rats in an experimental model of MS improved learning and memory impairment and alterations in the parameters of oxidative stress in the hippocampus [84].

#### 4.1.2. Effect on Alzheimer’s Disease

Alzheimer’s is one of the most common age-associated neurodegenerative diseases and leads to a significant deterioration in cognitive function. It is characterized by the formation of brain plates loaded with polypeptide β-amyloid and prominent tangles of tau protein. In vitro studies have shown that a water methanol (50:50 *v*/*v*) extract of saffron and crocin at different doses, is capable of inhibiting the fibrillogenesis of amyloid β-peptide [85,86]. Another mechanism involved in the anti-Alzheimer action of saffron is its inhibitory effect on the activity of acetylcholinesterase [87]. There are also clinical findings that have shown effectiveness in mild to moderate Alzheimer’s disease. Although more human clinical trials are needed, some studies have pointed to the neuroprotective character of saffron and its components, with effects being similar to those obtained with donepezil and nemantin in Alzheimer patients [7,88,89,90].

#### 4.1.3. Effect on Parkinson’s Disease

In addition to its therapeutic potential, crocetin has been shown to be potentially useful in reducing the risk of developing Parkinson’s disease. In a study conducted by Ahmad and coworkers [91] on hemiparkinsonian rats, the intraperitoneal administration of several doses of crocetin over 7 days protected the levels of thiobarbituric acid, a reactive substance in the substantia nigra. This indicates crocetin-protected, lipidic peroxidation induced-damage. Purushothuman and coworkers [92] studied the effects of a saffron extract (0.01% *w*/*v*), on dopaminergic cells in a mouse model with induced Parkinson, concluding that pretreatment with saffron protects many cells of the substantia nigra pars compacta.

#### 4.1.4. Effect on Cerebral Ischemia

The neuroprotective activities of saffron have been demonstrated in experimental models of cerebral ischemia. Saffron biocompounds are capable of attenuating all the alterations induced by ischemia, most likely due to its antioxidant properties. Vakili and coworkers [93], as well as other groups [94,95], showed that when saffron extract (15, 30, 60, and 120 mg/kg) was administered before the induction of focal cerebral ischemia, it improved the neurobehavioral outcome (grip strength, spontaneous motor activity and motor coordination) and protected the functionality of antioxidant defense systems (glutathione peroxidase, catalase and SOD). Moreover, Zheng and coworkers [77] observed that crocin reduced the oxidative damage in brain microvessels after the global model of cerebral ischemia. Safranal has been shown to protect the balance between oxidant and antioxidant systems, after an ischemia-reperfusion injury has been induced [72].

#### 4.1.5. Effect on CNS Tumours

Diffusion Pharmaceuticals LLC (Charlottesville, VA, USA) are developing a clinical trial (now in phase III) with *trans*-sodium crocetinate (TSC) as adjuvant in radiotherapy and chemotherapy treatments for resistant solid tumors such as glioblastoma, in which hypoxia is the result of its rapid growth. Cancer tumor cells thrive on hypoxia, because the resulting changes in the microenvironment offer resistance to treatment radiation and chemotherapy in hypoxic cancer cells. TSC re-oxygenates and promotes a greater diffusion of oxygen through the blood plasma that reaches the hypoxic tumor; this action converts TSC into a radiosensitizer [96,97].

#### 4.1.6. Age Related Macular Degeneration

Some studies on saffron provide important clues about its neuroprotective actions in age-related macular degeneration [98,99,100]. Such studies, both in vitro and in vivo, show that the administration of crocetin has protective effects against damage to the retina, inhibiting degeneration of photoreceptor and retinal dysfunction. This suggests that the mechanism is based on an inhibition of the increase in caspases 3 and 9 after retinal damage [101].

#### 4.1.7. Effect on Multiple Sclerosis (MS)

Antioxidant protection is a possible therapy for MS as ROS plays a key role in the initial and chronic phases of the disease. MS progresses with acute attacks and inflammation of the myelin. Much evidence indicates that MS plates suffer wide-ranging oxidative injury and that ROS-induced damage occurs from the earliest stages of neuroinflammation [102]. There are several treatments being trialed in clinical practice that use antioxidant diets which promise to be effective in the reduction of oxidative stress in MS [102,103].

Ghazavi and coworkers [79] indicated that saffron was able to reduce MS symptoms. After administering an ethanol extract of the spice, they observed a decrease in clinical symptoms and leukocyte infiltration in a model of experimental autoimmune encephalomyelitis (EAE).

Likewise, Deslauriers and coworkers [104] suggested that the glycosidic esters of crocetin might prevent demyelination and neurodegeneration. Such findings show that saffron may potentially prove useful in the treatment of MS through the inhibition of oxidative stress and the infiltration of leukocytes to the CNS.

#### 4.1.8. Effect on Nerve Damage Secondary to Diabetes Mellitus (DM)

One of the most severe complications of DM is the neuronal damage caused by a decrease in blood flow. More than half of diabetics develop some kind of nerve damage during the course of the disease [105]. In this respect, saffron has shown protective effects, presumably because of its antioxidant properties [40,106].

#### 4.1.9. Antidepressant and Anxiolytic Effects

Saffron and its metabolites have proven to be effective in different models of psychiatric disorders, including depression and anxiety. Various studies in clinical environments concluded that the daily administration of 30 mg of saffron could be useful in the management of depression when compared with imipramine or fluoxetine [107,108,109,110]. Indeed, some studies have demonstrated the usefulness of crocin tablets in conjunction with imipramine or fluoxetine [111]. A trial of 66 patients with anxiety-depressive disorder compared treatment with saffron (30 mg/day) or citalopram (40 mg/day) for 6 weeks. The results expressed in the Hamilton depression scale (HAM-D) and the Hamilton anxiety scale (HAM-A) showed no significant difference between the use of saffron and the drug and proposed saffron to be a potentially effective and tolerable treatment for this disorder [112].

Studies on the bioactive substances of saffron in depression indicate that the crocin acts by inhibiting the reuptake of dopamine and norepinephrine neurotransmitters, while safranal inhibits the reuptake of serotonin. There are in vivo studies suggesting inhibitory effects on the monoamine oxidases, MAO-A and MAO-B, enzymes responsible for the degradation of the neurotransmitters, as mentioned above, leading to an increase in their levels in the synaptic space and reducing depressive symptoms [113].

The satiating effect of ethanolic extract saffron (10% *w*/*v*) in decreasing the frequency of snacking events has also been studied by Gout and coworkers [70]. Similarly, Agha-Hosseini and coworkers [114] investigated saffron as a treatment for emotional disorders linked to premenstrual syndrome and found that using saffron (30 mg/day) was effective in relieving symptoms.

Some studies show that the aqueous extract (80, 160 and 320 mg/kg) and ethanol extract (800 mg/kg) of *C. sativus* reduces the signs of withdrawal symptoms of morphine in mice, suggesting an inhibitory effect of morphine dependence [115]. Likewise, a 7-day treatment with ethanolic and aqueous extracts (50, 100 and 200 mg/kg) and safranal (0.025, 0.05 and 0.1 mg/kg), attenuated the behavioral symptoms of neuropathic pain in a dose-dependent manner [116]. Other studies point to an improvement in memory and learning alterations induced by morphine with aqueous extract (50, 150 and 450 mg/kg) in mice [117].

### 4.2. Effect on the Cardiovascular System

Several studies highlight the potential effect of crocin in the treatment of diseases of the cardiovascular system. We have shown that the cardiovascular beneficial effects of aqueous crocetin extract from saffron stigma are related to the endothelial control of the vascular hyperreactivity of hypertension. Thus, crocetin is capable of improving the nitric oxide-endothelium-dependent vasodilation, which is damaged in hypertension [118]. At the same time, it presents anticontractile properties, reducing over-reactive arterial response, which is a hallmark of hypertension, by endothelium modulation [119].

In atherosclerosis, crocetin increases the diffusion of oxygen in the blood plasma, offsetting the decrease that occurs with increased cholesterol. In addition, it reduces the levels of cholesterol in the blood [120,121].

Many articles claim that crocetin could act as an antioxidant and have anti-inflammatory and antiapoptotic effects, thus providing a cardioprotective effect. A recent study by Wang and coworkers [122], in which crocetin was administered (50 mg/kg/day) during one week in a rat model of myocardial ischemia reperfusion injury, showed that crocetin pretreatment decreased serum level of pro-inflammatory factors (TNF-α) and increased anti-inflammatory factors (as IL-10). Additionally, it showed an anti-apoptotic protective effect, increasing anti-apoptotic protein Bcl-2 expression, endothelial nitric oxide synthase (eNOS) activity and nitrite (NO) serum levels, while also decreasing pro-apoptotic protein Bax RNAm expression. Furthermore, they observed that crocetin pretreatment enhanced endogenous antioxidant activity (superoxide dismutase) and diminished lipid peroxidation activity, indicating that crocetin can reduce the cardiac damage induced by oxidative stress.

In addition, numerous studies claim that crocetin increases the diffusion of oxygen and therefore the oxygenation of various tissues [96,123,124]. Yang and coworkers [125] demonstrated that the administration of crocetin (2 mg/kg) to experimental animals that had hemorrhagic shock, significantly improved survival and reduced apoptosis after resuscitation.

*trans*-Sodium crocetinate (TSC) is a new compound proposed for the treatment of ischemia after hemorrhagic shock, having been seen to increase the oxygenation of tissues and survival after hemorrhagic shock. As a result, there has been study of its possible application to reduce the damage caused by ischemia or hypoxia of neurological disorders [126]. Computer-simulated molecular models suggest that the interactions between the hydrophobic part of the molecule of TSC and water improves the passage of oxygen from the alveoli to the erythrocytes. In vitro measurements confirm a 30% increase in the rate of oxygen diffusion between erythrocytes and tissues as a result of altering the “structure” of water in blood plasma [123,124].

### 4.3. Effect on Liver Function

Hepatoprotective effects have been described in in vivo studies, whereby crocetin esters attenuated the activation of caspases, essential mediators of apoptosis or cell death, and the ratio between the proapoptotic bax and antiapoptotic BCL-2 (bax/Bcl-2) proteins, which reduces hepatotoxicity [127].

### 4.4. Antineoplastic Effect

Numerous studies suggest that the dietary intake of certain carotenoids may reduce the risk of developing some cancers, suggesting that they could exert beneficial actions in various tissues. Actions along these lines have also been attributed to saffron. Saffron aqueous extract inhibits the progression of different cancer types: gastric [128], colorectal [129], pancreatic [130], and bladder cancer [131], both in rats and cancer cells, reducing cell growth via apoptosis [132]. Recent studies with both in vitro and in vivo models indicate that saffron compounds, particularly crocin and crocetin, may have an anticancer effect in breast, lung, pancreatic and leukemic cells [5]. These constituents can reduce the cell proliferation at an early stage by inducing cell cycle arrest or at a later stage by inducing apoptosis. It must be noted that most of the investigations are aimed at studying the bioactivity of isolated metabolites of saffron, while the number of studies that look for anti-cancer properties in the natural form of saffron is much lower.

The mechanism of action is not clear, but its activity could be related to the antioxidant action of the esters of crocetin, which could affect the regulation of cell growth, modulate gene expression and the immune response [133]. Several studies point to saffron and its metabolites as activators of cellular apoptosis in cancer cells, while not affecting normal cells [66,128,134,135].

In animal models and cell culture systems, crocetin is reported to act on the growth cells in cancer by inhibiting nucleic acid synthesis, enhancing endogenous anti-oxidative system activity, inducing apoptosis and damaging the growth factor signaling pathways [136].

The results of studies involving cancer cells vary greatly. According to Abdullaev [137], these differences may be due to the existence of different types of cell receptors, the rate of intracellular transport, differences in the amount of compound used, different methods of extraction and determination of any cytotoxicity. Some of these effects are the following:

- In dermatological tumors in mice, the oral administration of saffron inhibits the formation of papillomas and simultaneously decreases their size [138].
- There are studies, both in vitro and in vivo, with pancreatic tumor cells which indicate that crocetin and its esters could have a potent antitumor action via the inhibition of apoptosis [139]. When saffron extract (300 mg/kg), crocin (200 mg/kg) and crocetin (100 mg/kg) were tested on prostate cancer, the tumor size decreased. In particular, crocetin, reduced tumor mass by 75–85% [140]. In colorectal cancer cell lines (HCT-116, SW-480 and HT-29), crocetin esters inhibited the growth of malignant cells, while not affecting the growth of normal cells [141].

### 4.5. Anti-Adiposity Effects

An in vivo study carried out by Sheng and coworkers [142] suggests a lipid-lowering effect induced by the crocetin esters. The results showed that, although the esters of crocetin did not directly block the absorption of cholesterol, they could inhibit the activity of pancreatic lipase. The effects of the uptake of lipids and decrease in obesity in rats have been observed, as well as an improvement in the lipid profile as a result of saffron aqueous extracts (20, 40, and 80 mg/kg) and crocetin esters (25 to 100 mg/kg/day) [61,143]. In addition, it has been shown that crocetin is capable of preventing the pro-inflammatory state that occurs in obesity, by decreasing the expression of TNF-α and increasing adiponectin, in adipose tissue of fructose-fed rats [144]. In another study, also involving rats, the administration of crocetin prevented the accumulation of visceral fat and insulin resistance that a hyper-caloric diet-induced, without the consumption of feed being affected [145].

### 4.6. Other Effects

Some studies attribute anti-inflammatory activity to saffron [146], in addition to anticonvulsant [71], antihistamic, anti-asthmatic, genoprotective, antitussive, gastric mucosa protective and sexual dysfunction effects [11]. Table 1 summarizes the bioactivity in humans demonstrated in clinical trials.

## 5. Saffron as a Functional Food. Dosage for Nutritional Intervention

Many foodstuffs have traditionally been associated with beneficial and even medicinal effects. While saffron has been used for thousands of years as a spice, it is notable both for its nutritional qualities and its medicinal effects [1,8,58,147].

Many terms are ascribed to food products that supposedly have health benefits: functional foods, designer foods, nutraceuticals and pharmafoods. However, if we consider only the legal definition, they can only be referred to as new foods, medical foods, dietary supplements and herbs or medicinal plants [148]. Interestingly, the journal *Nature Biotechnology* was already describing the concept of “agriceuticals” in 1999 [149].

Research into the effects of saffron and its components is necessary to establish suitable doses and its safety, this should lead to more widespread use of the spice for its functional or bioactive properties.

The correct dose of saffron is essential to ensure safety and beneficial effects. Most of the studies are carried out in vitro and in vivo and it is difficult to know human doses. In some cases, the doses mentioned are acceptable for administration in food, but in other cases, especially in studies involving safranal, the doses are unacceptable, considering that they will be administered as part of the diet and not as a treatment [11].

The safety of saffron as a spice has been established by its use as a food additive to provide color and flavor over centuries, while traditionally extracts and tinctures of saffron have been used in the treatment of diseases with no toxic side effects [59].

An extract from the 1987 European Commission monograph about saffron discussed the dangers of dosages in food: “to a daily maximum dose of 1.5 grams there has not been any risk documented. Lethal dose is 20 g and the abortive dose, 10 g, because as such it was employed in the past due to its stimulating action on the smooth muscle of the uterus. 5 g daily dose can already cause intoxication accompanied by vomiting, bloody diarrhea, hematuria, skin hemorrhages in nose, lips and eyelids, vertigo and dulling. The skin and mucous membranes take a yellowish colour similar to jaundice”.

However, more recently, and as a result of the use of saffron in clinical trials, numerous studies have been conducted to assess the possible toxicity of saffron in experimental animal and in vitro studies [150], and no important hematological and biochemical parameters changes have been observed, and none that might suggest its toxicity [151].

High doses of saffron should be avoided during pregnancy. Quantities of more than 5 g, far greater than that used in food, can act as a uterine stimulant and have abortifacient effects [152]. The spice has had its safety evaluated in a study that involved healthy volunteers being divided into three groups and taking tablets equating to 400 mg daily for 7 days. The study found that while saffron may change some hematological and biochemical parameters, any alterations were within normal ranges and involved no clinical disorders [151].

Several experiments have demonstrated that high doses of saffron, as used in rats, do not imply high toxicity levels [153]. A study assessed the acute toxicity of saffron in 32 pregnant mice administered saffron orally via a probe, representing 500, 1000 and 2000 mg/kg/day of saffron for three weeks, to four groups of mothers. A normal saline solution was supplied to the control group. Nephrotoxicity and hepatotoxicity were evaluated in the offspring. In a sub-acute assessment, it was seen that saffron is a safe spice with no toxic effects on the liver, although the administration of high doses of saffron to mothers could damage the kidneys of the newborn [154].

The mean lethal dose (LD50) of saffron is 200 mg/mL in vitro and 20.7 g/kg in animal studies [155]. The joint administration of the aqueous extract of saffron and safranal reduces the effects of the acute and sub-acute toxicity of safranal. It reduces mortality and the toxic effects of safranal on specific biochemical markers. Based on these results, the consumption of an aqueous extract of saffron spice, rather than safranal alone, is to be recommended [156].

The interaction of saffron with other drugs has not been studied. It is known that crocetin binds strongly to albumin serum, but the transport of drugs in plasma has not yet been evaluated [54,157].

Daily 30 mg doses used in clinical trials into the effects on depression have shown no difference in tolerance compared with a placebo, although there were adverse effects including nausea, vomiting, and headaches [158,159].

## 6. Conclusions and Future Perspectives

New studies are needed to understand the physiological behavior of saffron compounds after oral ingestion, as well as the absorption, distribution, metabolism and excretion (ADME) of the compound’s saffron. In addition, study of the spice’s bioaccessibility and bioavailability is essential to determine the pharmacological and toxicological profile of these compounds.

Although there are studies on the pharmacokinetics of crocin administered orally, studies that determine the pharmacokinetics of saffron as a spice are still lacking. In most publications that study the bioactivity of saffron, there is no characterization of the initial sample, and so the contents of its major metabolites are unknown. There are many studies that do not clearly describe the methods used to prepare the various forms of saffron. This is an issue of great importance since it most likely affects the concentration and absorption of the metabolites.

More studies that determine the pharmacokinetics of saffron and glycosidic esters of crocetin are needed, but require proper characterization of the samples. This knowledge is necessary to prepare a therapeutic diet with this spice. Nowadays, there is a growing interest in the use of special diets that include culinary herbs or spices to help in the treatment of different diseases. The antioxidant and anti-inflammatory nature of some spices has been known for a long time to protect against chronic diseases. Therapeutic diets using saffron are a result of the much lower incidence of certain neurodegenerative diseases in the Asian subcontinent, where people consume large amounts of spices, as opposed to in the western world. The idea of preventive therapy for neurodegenerative diseases using spices is based on evidence obtained during the past 10 years concerning the use of nutraceuticals derived from spices.

The potential healthy effect of saffron will depend on the amount consumed and its bioavailability. Although the quantities of saffron consumed varies considerably between different regions and types of culinary diet, it is normally used in small quantities as seasoning, in order to give color, flavor and aroma to food. The spice contains high concentrations of bioactive compounds that can contribute to its beneficial effects. As mentioned above, there is little knowledge about their bioavailability and they may be affected by various chemical and biochemical factors. The bioavailability of spices should be subject to epidemiological studies so as to confirm the beneficial effects of their intake.

## Figures and Tables

**Table 1:** Summary of the clinical trials found in the literature. The supplied agent is indicated, if an analysis of its composition has been carried out, time in weeks of application, dose in mg/day, demonstrated bioactivity and reference.

Agent Supplied	Analysis Comp.	Time (Week)	Dose (mg/day)	Bioactivity	Ref.
Saffron ethanol (80%) extract	Bad analysis	16	30	Alzheimer‘s disease	[88]
Saffron ethanol (80%) extract	Bad analysis	22	30	Alzheimer‘s disease	[89]
Saffron ethanol (80%) extract	Bad analysis	48	30	Alzheimer‘s disease	[90]
*trans* Sodium crocetinate	Yes	undefined	0.25	Glioblastoma	[96]
Saffron	Yes	12	20	Macular degeneration	[98]
Saffron	No	56	20	Macular degeneration	[99]
Saffron	No	12	20	Macular degeneration	[100]
Saffron aqueous extract (SAE) and crocin (CR)	Yes	12	SAE: 30 CR: 30	Schizophrenia	[106]
Saffron ethanol (80%) extract	No	6	30	Depression	[107]
Saffron ethanol (80%) extract	No	6	30	Depression	[108]
Saffron ethanol (80%) extract	No	6	30	Depression	[109]
Saffron ethanol (80%) extract	No	6	30	Depression	[110]
Crystallized crocin	No	4	30	Depression	[111]
Saffron	No	6	30	Depression	[112]
Saffron ethanol (80%) extract	No	8	30	Premenstrual syndrome	[114]
Saffron	Yes	12	50	Lipid Profile	[121]
